# Support for the Transmission-Clearance Trade-Off Hypothesis from a Study of Zika Virus Delivered by Mosquito Bite to Mice

**DOI:** 10.3390/v11111072

**Published:** 2019-11-18

**Authors:** Kathryn A. Hanley, Sasha R. Azar, Rafael K. Campos, Nikos Vasilakis, Shannan L. Rossi

**Affiliations:** 1 Department of Biology, New Mexico State University, Las Cruces, NM 88003, USA; khanley@nmsu.edu; 2 Department of Pathology, The University of Texas Medical Branch, Galveston, TX 77555-0609, USA; srazar@utmb.edu; 3 Department of Microbiology and Immunology, The University of Texas Medical Branch, Galveston, TX 77555-0609, USA; rkkroonc@utmb.edu; 4 Center for Biodefense and Emerging Infectious Diseases, University of Texas Medical Branch, Galveston, TX 77555-0609, USA; 5 Center for Tropical Diseases, University of Texas Medical Branch, Galveston, TX 77555-0609, USA; 6 Institute for Human Infection and Immunity, University of Texas Medical Branch, Galveston, TX 77555-0610, USA

**Keywords:** Zika virus, *Aedes albopictus*, evolution of virulence, transmission–clearance trade-off, within-host dynamics, flavivirus, arbovirus, A129 mice

## Abstract

Evolutionary theory indicates that virus virulence is shaped by a trade-off between instantaneous rate of transmission and duration of infection. For most viruses, infection is curtailed by immune clearance, but there are few empirical tests of the transmission–clearance trade-off hypothesis. We exposed A129 mice to bites from groups of 1, 2–4, or 6–9 *Aedes albopictus* mosquitoes infected with Zika virus (ZIKV). We predicted that a higher number of infectious mosquito bites would deliver a higher total dose of the virus, and that increasing dose would result in earlier onset, higher magnitude, and shorter duration of viremia, as well as a more robust neutralizing antibody response. We found that increases in the number of mosquito bites delivered resulted in significantly different virus replication dynamics with higher, earlier peak titers. All mice experienced a transient weight loss following infection, but the nadir in weight loss was delayed in the mice that received the highest number of bites. Viremia persisted past the period of measurement in this study, so we did not capture its duration. However, the association at the level of the individual mouse between the estimated virus dose delivered and neutralizing antibody titer was remarkably strong, supporting the transmission–clearance trade-off hypothesis.

## 1. Introduction

The fitness of a virus in a host species is determined, in part, by its virulence in that species. Explaining and predicting the evolution of virulence has been a goal of evolutionary biologists for decades [1,2,3,4,5,6]. Theory on the evolution of virulence rests on the premise that pathogen fitness is maximized by optimizing the trade-off between instantaneous pathogen transmission and duration of infection [2,7]. In the majority of theoretical studies, host mortality has been invoked as the mechanism that regulates duration of infection, i.e., [2,8,9,10]. However, most viruses do not kill their hosts [7,11], and infections are cleared by the immune response [7,11]. For these viruses, the key trade-off shaping virulence is between transmission and immune-mediated clearance [12].

While the term virulence is often used synonymously with harm to the host, such harm results from a complex combination of direct, pathogen-mediated effects and immune-mediated damage [13]. The current study focuses on Zika virus (ZIKV), a member of the genus *Flavivirus*. Magnitude of replication (i.e., peak virus titer) of ZIKV, and of flaviviruses generally, tends to correlate with disease severity, although disease itself is largely mediated by the immune response to infection [14,15,16,17,18,19]. Moreover, an increasing magnitude of viremia has been shown to increase instantaneous transmission rate for many flaviviruses, including ZIKV (reviewed in [20], see also [21,22,23,24]). Thus, we define virulence here simply as the level of replication of the pathogen within the host.

The transmission–clearance hypothesis predicts that, as the magnitude of infection increases, instantaneous transmission will increase, but the rapidity with which that infection will be cleared will also increase. However, empirical studies of this process are scarce. We have previously investigated this trade-off by reviewing the literature on the intra-host dynamics of arthropod-borne viruses (arboviruses) during experimental infection of vertebrate reservoir hosts to determine whether the magnitude of infection was inversely related to duration of infection, and, indeed, it was in the majority of studies [20]. Furthermore, the dose of virus delivered had a significant impact on within-host dynamics, with higher doses resulting in higher-magnitude, shorter-duration infections [20]. We then used a dynamical model to contrast the effect of low-magnitude, long-duration viremia, termed a “tortoise” strategy in a nod to Aesop’s fable, in a vertebrate reservoir host, and a “hare” strategy of short-duration, high-magnitude viremia. Arboviruses that adopted a tortoise strategy had higher rates of persistence in both reservoir host and vector populations [20].

A limitation of the studies reviewed in [20] was that, in almost all cases, virus was delivered to the host via needle. Arbovirus replication, immunogenicity, pathogenesis, and transmission differ significantly when virus is delivered by mosquito bite versus a needle [25,26,27,28,29]. Ben-Shachar and Koelle [30] captured this natural mode of infection when they analyzed within-host dynamics of dengue virus (DENV) in a human clinical cohort, in order to evaluate the transmission–clearance trade-off hypothesis. Their within-host simulation models demonstrated that a transmission–clearance trade-off occurs and, further, that this trade-off selects for the evolution of intermediate DENV virulence. However, as this was a study of infected individuals in the clinic, it was not possible to infer how many mosquito bites initiated infections, what the dose of virus delivered by those mosquitoes was, or to what magnitude the virus replicated early in infection, before the onset of symptoms.

In the current study, we tested the transmission–clearance trade-off hypothesis by delivering ZIKV to interferon (IFN) type I-deficient A129 mice via infected *Aedes albopictus* mosquitoes. A129 mice were chosen as a model for this study because of their susceptibility to ZIKV, even though such susceptibility comes at the price of type-I IFN, a key mediator of the innate immune response to ZIKV infection [31]. Nonetheless, several other host pathways have recently been shown to restrict ZIKV replication, including the cellular stress response, the NMD pathway, and reticulophagy [31]. Moreover, the ability of A129 mice to marshal a neutralizing antibody response is well-established [32]. Thus, we anticipated that ZIKV replication would trigger relevant immune responses in this system.

Mice were exposed to cartons containing different numbers of infected mosquitoes. Subsequent to feeding, saliva was collected from engorged mosquitoes by forced salivation and the dose of virus delivered in each saliva sample was quantified. We predicted, based on this hypothesis, that a higher number of infectious mosquitoes would deliver a higher total dose of virus, and that increasing dose would drive earlier onset of viremia, higher magnitude of viremia, shorter duration of viremia, and a more robust neutralizing antibody response by the mice. Most of these predictions were borne out, offering key insights into the interplay between the dose of virus delivered by mosquitoes, subsequent intra-host virus dynamics, and neutralizing antibody responses to infection.

## 2. Materials and Methods 

### 2.1. Animals, Study Design and Ethics Statement

All A129 mice were purpose-bred at University of Texas Medical Branch (UTMB) for this study and maintained in sterile caging supplemented with food and water *ad libitum*. Mice of both genders were exposed to infected mosquitoes at 4 weeks of age. All experiments were performed in full compliance with the guidelines established by the Animal Welfare Act for the housing and care of laboratory animals and conducted as laid out in University of Texas Medical Branch (UTMB) Institutional Animal Care and Use Committee-approved protocol no. 1807054 (issued: 01 July 2018). Mice were anesthetized via intraperitoneal injection with Ketamine-Xylazine (K:100 mg/kg, X:10 mg/kg). Anesthetized animals were placed onto 0.5 L containers containing either 1, 2, 5, or 10 ZIKV-infected *Ae. albopictus* (Galveston, F8) (detailed below) and mosquitoes were allowed to feed until engorgement. Any un-engorged mosquitoes were removed from the study. Mice were weighed daily and monitored for signs of ZIKV disease for 14 days. Any mice that showed signs of neurological disease or lost more than 20% of their initial weight were euthanized by carbon dioxide asphyxiation. Blood was removed from the retro-orbital sinus of individual mice on alternating days during the period from day 1–5 post mosquito feeding, and at day 14 post-feeding. Blood was clarified by centrifugation and sera were transferred to new tubes for titration.

### 2.2. Mosquitoes

Female *Ae. albopictus* from a Galveston, Texas colony (F8) were utilized in these experiments. Mosquitoes were housed in a 27 ± 1 °C incubator at a 16:8 light:dark photoperiod with 80% ± 10% relative humidity, fed 10% sucrose ad libitum, and maintained, sampled, and processed as described previously [21]. Two days post-eclosion, each mosquito was injected intrathoracically with 300 focus-forming units (FFU) of the PRVABC59 (Puerto Rico, 2015) strain of ZIKV in a volume of 100 nanoliters. Seven days later, groups of 1–10 mosquitoes were separated into 0.5 L cardboard cartons overlayed with mesh, starved of sugar overnight, and used to expose individual mice to ZIKV, as described above. To determine the titer of ZIKV salivated into these mice, mosquitoes that engorged on the mice were maintained for two days, at which point legs and wings were removed into individual tubes (to confirm infection) and mosquitoes were restrained on mineral oil. Proboscis were inserted into micropipette tips filled with 10 µL of FBS, and mosquitoes were allowed to salivate for 30 min. Sera and saliva were titrated on Vero cell monolayers and processed as described below.

### 2.3. Cell Lines and Viruses

Vero cells (CCL-81) were purchased from the American Type Culture Collection (Bethesda, MD, USA) and maintained in DMEM supplemented with 5% FBS and penicillin/streptomycin (P/S; 100 Units/mL and 100 µg/mL respectively) at 37 °C with 5% CO_2_. ZIKV strain PRVABC59 was provided by the World Reference Center for Emerging Viruses and Arboviruses (WRCEVA, Galveston, USA) at fourth passage in Vero cells. Two subsequent amplifications from this lyophilized stock were conducted in Vero cells to produce the stock utilized in this study.

### 2.4. Titrations

Virus samples were assayed by 10-fold serial dilution in DMEM on Vero cell monolayers. After 1 h at 37 °C, wells were overlaid with 0.8% methylcellulose in DMEM. Following 3 days incubation at 37 °C, the overlay was removed and monolayers were rinsed twice with sterile DPBS, and fixed for 1 h at room temperature in ice-cold methanol:acetone (1:1). Detection of virus was conducted via focus-forming assay, as detailed below.

### 2.5. Focus Forming Assay

Focus-forming assays were performed as previously described [21], with modifications. Viral dilutions or clarified mosquito homogenates were inoculated onto Vero cell monolayers on 12- or 96-well plates, respectively. Following a 3 day infection, plates were washed and fixed as described above, and stained using a mouse hyperimmune polyclonal anti-ZIKV primary antibody (WRCEVA), and HRP-labeled goat anti-mouse secondary antibody (KPL, Gaithersburg, MD, USA). Detection was performed using an aminoethylcarbazole (AEC) solution (Enzo Diagnostics, Farmingdale, NY, USA) prepared according to manufacturer’s protocol. 

### 2.6. Plaque Reduction Neutralization Test Assay

Neutralization/seroconversion of sera collected at day 14 post-feeding was determined utilizing a standard 80% plaque reduction neutralization test (PRNT_80_) as previously described [33,34]. The neutralizing titer was represented as the reciprocal of the highest dilution of serum that inhibited 80% of foci (PRNT_80_). Samples scored below the limit of detection for a single plate (<1:20) were considered negative. Vero-passaged (Vero-1, C6/36-2, Vero-4) ZIKV FSS13025 isolate (WRCEVA) was utilized as the reference strain. 

## 3. Results

### 3.1. Mosquito Feeding Success, Detection of Virus in Saliva, and Grouping of Mice for Analysis

We used A129 mice, which lack the receptor for type I interferon, to investigate ZIKV intra-host dynamics, because we have previously shown that this strain of mice is very susceptible to ZIKV infection. [35]. To achieve high levels of replication coupled to low levels of mortality [35], we exposed four-week old mice to cartons containing either 1, 2, 5 or 10 *Ae. albopictus* that had been intrathoracically injected with ZIKV. All mosquitoes injected with ZIKV contained detectable virus in their thorax. Of the 108 mosquitoes used, 79 (73%) became engorged. Moreover, of the 79 engorged mosquitoes, 49 (62%) produced detectable virus in the saliva. For these 49 mosquitoes, the range of ZIKV titer per saliva sample for individual mosquitoes with detectable virus in the saliva was 0.3–3.0 log_10_FFU.

To accurately reflect the actual number of bites received, mice that were exposed to cartons holding two mosquitoes but were fed upon by a single mosquito were reassigned to the “one mosquito” analysis group. Only two mice were fed upon by two mosquitoes, so these mice were grouped with those mice that had been fed upon by three to four mosquitoes (no mice were fed upon by five) in a group called “two to four mosquitoes”. No mice were fed upon by a full complement of ten mosquitoes, instead, the range of mosquitoes that fed in the ten mosquito cartons was six to nine, so this treatment was renamed “six to nine mosquitoes” (Table 1).

Of the 24 mice used in the experiment, three that could not be shown to have been exposed to ZIKV were excluded from further analysis: mouse number 4 was exposed to a single mosquito that did not feed, and this mouse produced no detectable viremia, nor did it seroconvert by day 14 post-infection (Table 1); mice numbers 9 and 11 were exposed to two mosquitoes each, of which a single mosquito fed in each case, neither of the fed mosquitoes produced detectable virus in the saliva and neither of the mice produced viremia or seroconverted (Table 1). Two additional mice were notable. Mouse number 3 was fed upon by one mosquito with virus in saliva but failed to become viremic by day 5 post-feeding or to seroconvert by day 14 post-feeding (Table 1); this mouse was retained in all analyses but excluding it generally enhanced statistical significance in comparisons among treatments. Additionally, mouse number 5 was fed upon by a single mosquito that did not produce detectable virus in its saliva, nonetheless this mouse became viremic.

### 3.2. Intra-Host Virus Dynamics

**Figure 1:**
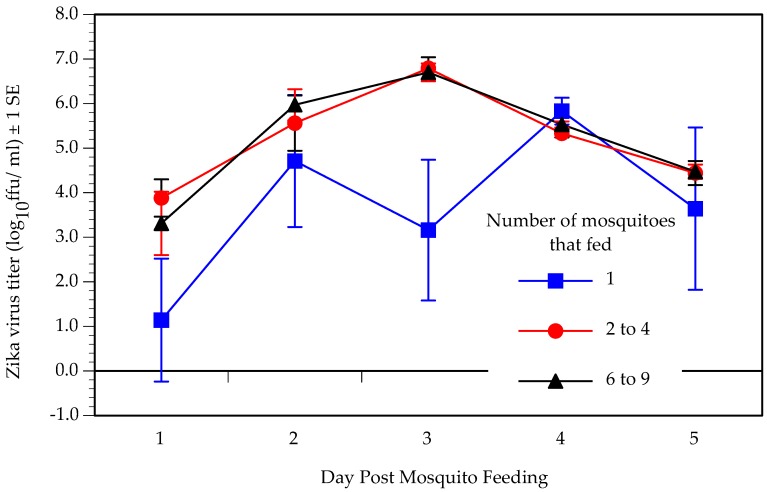
Mean Zika virus titer on designated day post-feeding in A129 mice fed upon by one (*n* = 7), two to four (*n* = 8), or six to nine (*n* = 6) infected *Aedes albopictus* mosquitoes.

We predicted that dynamics of within-host replication would shift with increasing number of infectious mosquito bites. Figure 1 shows the replication of ZIKV in the three treatment groups. A two factor ANOVA revealed that both number of mosquitoes that fed (DF = 2, F = 4.29, *p* = 0.02) and day post-infection (DF = 4, F = 8.64, *p* < 0.0001) had a significant effect on virus titer, but there was no significant interaction between the two factors (DF = 8, F = 1.76, *p* = 0.13). Post-hoc t-tests showed that mice fed upon by a single mosquito experienced a significantly lower level of virus replication than mice fed upon by two to four or six to nine mosquitoes, although the latter two groups did not differ from one another. 

**Figure 2:**
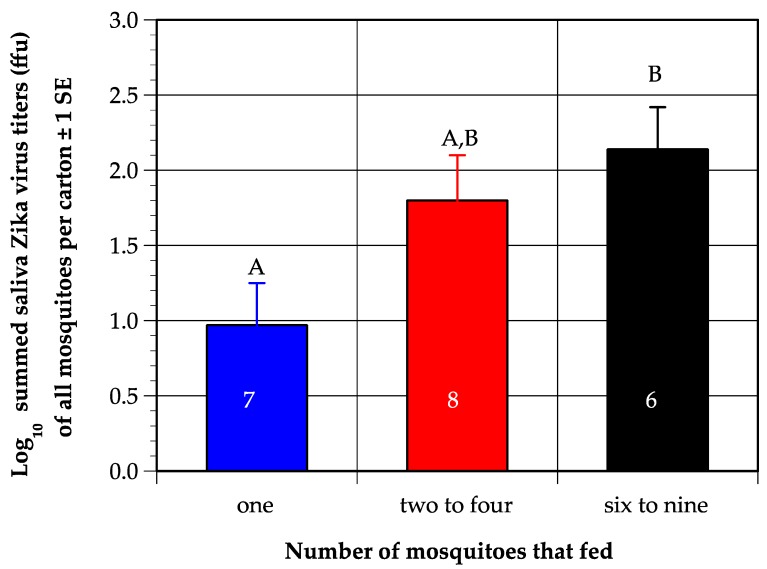
Mean of the summed titer of Zika virus (ZIKV) in saliva from each carton of mosquitoes in each designated treatment group; numbers inside bars indicate number of replicates. Letters above bars represent the results of post-hoc student’s t tests; values that do not share a letter are significantly different. Numbers inside bars indicate number of mice per treatment.

Although it was initially puzzling that viremia in mice fed upon by two to four mosquitoes did not differ from viremia in mice fed upon by six to nine mosquitoes, a comparison of the total virus in the saliva of all mosquitoes combined in each carton (termed summed virus), collected via forced salivation, revealed that the mean summed virus produced by groups of two to four mosquitoes (1.8 log_10_ffu) did not differ substantially from groups of six to nine mosquito (2.1 log_10_ffu), whereas both groups produced about tenfold more virus that single mosquitoes (1.0 log_10_ffu) (Figure 2). The difference among the three groups was significant (one-way ANOVA on log-transformed values, DF = 2, *p* = 0.03), and post-hoc t-tests confirmed the summed saliva titer of single mosquitoes differed significantly from six to nine mosquitoes, while the two-to-four mosquito group did not differ significantly from the other two treatments.

We predicted that magnitude, or peak titer, of viremia would increase with increasing virus dose. The median peak titer showed a marginally significant difference among the three treatment groups (Kruskal–Wallis test, DF = 2, *p* = 0.05) and a Wilcoxon post-hoc test showed that peak titer was significantly lower in mice fed upon by one mosquito (5.5 log_10_ ffu/mL) than mice fed upon by six to nine mosquitoes (6.4 log_10_ ffu/mL), while the peak titer in mice fed upon by two to four mosquitoes (6.3 log_10_ ffu/mL) was intermediate to, and did not differ significantly from, the other two groups. Because of the similarity in ZIKV dose delivered between the groups of two to four and six to nine mosquitoes, we also combined these two groups into a single group of >1 mosquito, and the median peak titer was significantly lower in the 1 mosquito group than the >1 mosquito group (Mann–Whitney U test, DF = 2, *p* = 0.04).

We also predicted that the lag to viremia and lag to peak viremia would decrease with increasing virus dose. All but two of the mice were viremic on the first day that they were sampled; of the two that were not, one became viremic by day 3 post-feeding and one had not become viremic by day 5 (Table 1). Both were fed upon by a single mosquito. Analysis of lag to peak titer was complicated by the fact that one cohort of mice was sampled on days 1, 3 and 5, and a second cohort on days 2 and 4. To render these data comparable, we coded peak titer as early if it was reached by day 3 post-feeding and late if it was reached after day 3. To meet the assumptions of a contingency table analysis, the mice fed upon by two to four and six to nine mosquitoes were combined into a >1 mosquito treatment group. Significantly fewer (33% of six) mice fed upon by 1 mosquito reached their peak titer early than mice fed upon by >1 mosquito (86% of 14) (Fisher’s exact test, *p* = 0.04).

### 3.3. Mouse Survival and Weight Changes

**Figure 3:**
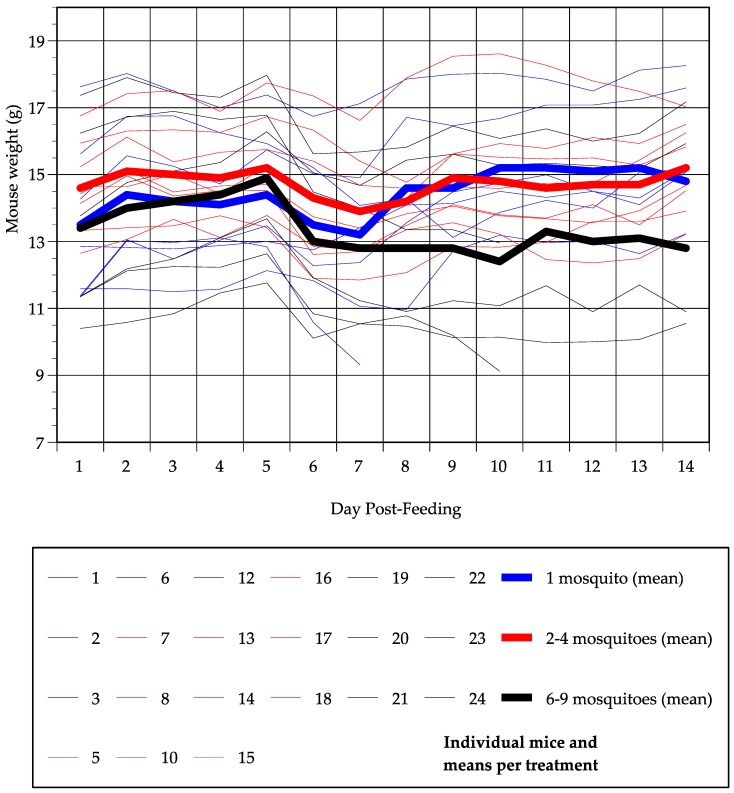
Weight of each mouse (thin lines) and mean weight per treatment group (thick lines) on each day post-feeding for each of the three mosquito feeding groups. Blue indicates mice fed upon by one mosquito, red indicates two to four mosquitoes and black indicates six to nine mosquitoes.

Three mice died over the course of the 14-day monitoring period; two of these were fed upon by six to nine mosquitoes (mice numbers 22 and 24) and one was fed upon by a single mosquito (mouse number 10). Total weight change was quantified as ((weight on day 14 − weight on day 1)/weight on day 1). For mice that died, the last available weight measurement was used in place of the day 14 measurement. The majority of mice that survived gained some weight by day 14 post-feeding (Figure 3), and there was no significant difference in total weight change among the three treatment groups (ANOVA, DF = 2, *p* = 0.15) or between mice fed upon by 1 or >1 mosquito (*t*-test, DF = 19, *p* = 0.12). However, all mice did show a dip in weight between day 1 and day 14 (Figure 3). There was no significant difference among treatment groups in normalized nadir weight ((lowest weight during monitoring period − weight on day 1)/weight on day 1) (ANOVA, DF = 2, *p* = 0.72), with mice fed upon by one, two to four, or six to eight mosquitoes, losing 5%, 6% and 9% of day 1 body weight on average, respectively. There was, however, a significant difference in the day at which that nadir occurred (ANOVA, DF = 2, *p* = 0.02). On average, mice fed upon by one mosquito dropped to their lowest weight 6.6 days post-feeding, mice fed upon by two to four mosquitoes dropped to their lowest weight 7.1 days post-feeding and mice fed upon by six to nine mosquitoes dropped to their lowest weight 9.3 days post-feeding; post-hoc t-tests revealed that the day of nadir weight differed significantly between mice fed upon by one and six to nine mosquitoes, and between two to four and six to nine mosquitoes, while there was no difference between one and two to four mosquitoes. There was a negative relationship between the day of nadir weight and normalized nadir weight (i.e., at later days post-feeding, the weight loss was greater), but this relationship was not significant (correlation = −0.40, *p* = 0.07). Notably, mice in the one mosquito treatment and the six to nine mosquito treatment started at a similar weight, and mice in the six to nine mosquito treatment actually weighed slightly more than mice in the one mosquito treatment by day 5 post-feeding, but, by day 8 post-feeding, the mice in the six to nine mosquito treatment had dropped to a lower mean weight than mice in the one mosquito treatment and this difference persisted until day 14.

### 3.4. Seroconversion

We predicted that a higher number of infectious mosquito bites would elicit more neutralizing antibodies. For this analysis, PRNT_80_ values lower than the limit of detection (20) were arbitrarily set to 10. Moreover, for mice that died prior to day 14, blood was collected on the day of euthanasia for PRNT analysis. One sample (from a mouse fed on by 2–4 mosquitoes) was not obtained.

**Figure 4:**
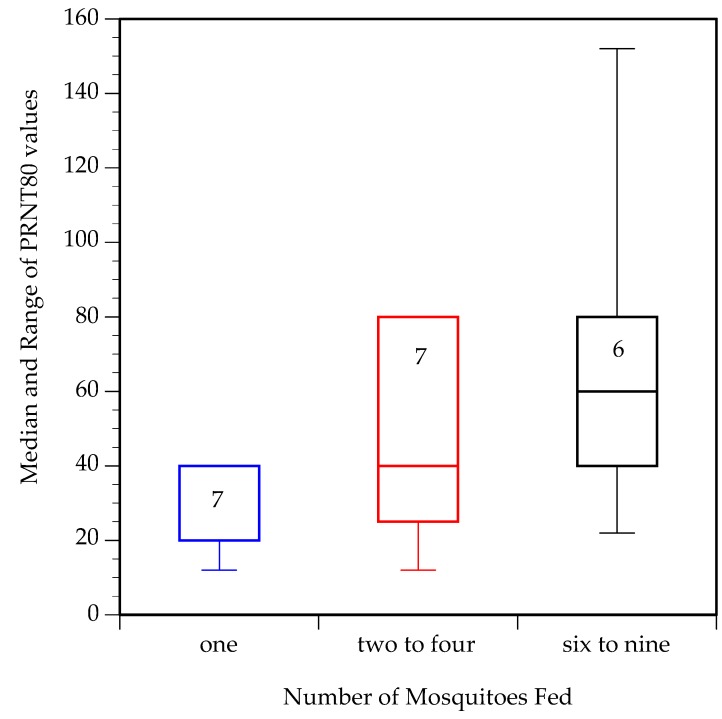
Median and range of plaque reduction neutralization test (PRNT)_80_ values on day 14 post-feeding from mice in specified treatment groups. Numbers inside bars indicate number of mice.

**Figure 5:**
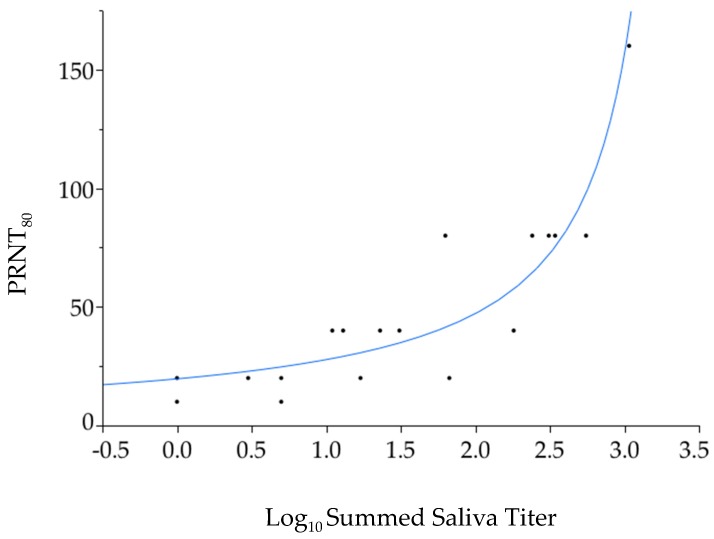
Association between summed mosquito saliva Zika virus titer estimated via forced salivation and mouse PRNT_80_ values measured 14 days after mosquito feeding.

PRNT_80_ values (Table 1, Figure 4) were not significantly different among the three treatment groups (Wilcoxon test, DF = 2, *p* = 0.11), but were significantly lower in mice fed upon by one mosquito than mice fed upon by more than one mosquito (Mann–Whitney U test, DF = 1, *p* = 0.048). Across all mosquito feeding groups, a general linear model found a strongly significant, positive association between the summed virus titer in mosquito saliva per mouse and PRNT_80_ (Figure 5, DF = 1, *p* = 0.008). This association remained significant when the one mouse that produced the maximum PRNT_80_ value of 160 was excluded (*p* = 0.04). The same analysis did not find a significant association between PRNT_80_ and peak titer in the mouse (*p* = 0.22) or actual number of mosquitoes that fed upon the mouse (*p* = 0.19).

## 4. Discussion

To test the transmission–clearance trade-off, we subjected A129 mice, a model that we initially developed to investigate ZIKV replication [35], to bites from different numbers of *Ae. albopictus* mosquitoes infected with ZIKV. We have previously demonstrated that *Ae. albopictus* is a competent vector for ZIKV [21]. Because of variation in mosquito feeding, the initial treatment groups, consisting of 1, 2, 5 or 10 mosquitoes, were reconfigured into 1, 2–4 or 6–9 mosquitoes for analysis. The amount of virus delivered was estimated by allowing mosquitoes to salivate into buffer two days post-feeding on the mice. The estimated dose of virus by the complete cohort of mosquitoes in a carton differed substantially between the one mosquito treatment, which delivered approximately 10 ffu, and two to four or six to nine mosquito treatments, both of which delivered approximately 100 ffu. These data reveal that ZIKV transmission is possible with as few as one feeding mosquito in a highly susceptible model. However, the dose estimated by forced salivation is likely substantially lower than the dose delivered during feeding. Styer et al. [36] demonstrated that the dose of West Nile virus (WNV) delivered by *Culex* mosquitoes during feeding was 600 times higher than the dose delivered via capillary. If this is also true for ZIKV delivered by *Ae. albopictus*, the dose of ZIKV delivered in this study by mosquitoes falls into the range of typically used needle-delivered inoculations of ZIKV in murine studies of 3–5 log_10_ ffu/mouse. Alternatively, as has been demonstrated for other flaviviruses (reviewed in [37]), mosquito saliva may enhance ZIKV infection and lower the minimum mouse infectious dose via the “inflammatory niche” created by the immune response to saliva [38]. Hastings et al. have recently shown that a protein in mosquito saliva, NeSt1, can enhance ZIKV infection in mice by stimulating the production of pro-IL-1β, CXCL2, and inducing neutrophil activation and macrophage recruitment at the feeding site [39].

We predicted that increasing the dose of virus delivered would reshape virus replication dynamics and increase magnitude (peak titer) of viremia, shorten the lag to peak viremia, and shorten duration of viremia. We found that virus replication was muted in mice fed upon by a single mosquito relative to mice fed upon by two to four or six to nine mosquitoes. Moreover, lag to peak viremia was shorter and peak viremia was higher as mice received more mosquito bites. Because infections persisted beyond the five-day window of measurement in this study, we were not able to quantify duration of viremia. Replication dynamics of ZIKV in the A129 mice following delivery by mosquito bite were generally similar to what we have observed in A129 mice of the same age that were infected with ZIKV intra-peritoneally (IP) [35], save that peak titer occurred a day earlier in mice injected IP.

For flaviviruses, the severity of host disease is often correlated with the magnitude of virus replication. As expected from our prior work [29], relatively few mice died following ZIKV infection, but all mice that survived experienced a transient dip in weight. However, mice fed upon by more than one mosquito did not experience a significantly greater loss of weight at their nadir weight than mice fed upon by a single mosquito, even though the former group of mice experienced a higher, earlier peak in viremia. Interestingly, there was a significant difference among treatment groups in the lag to nadir weight, with mice fed upon by a single mosquito reaching their lowest weight three days earlier than mice fed upon by six to nine mosquitoes. Moreover, mice fed upon by six to nine mosquitoes did not recover weight to the same extent as the other two treatment groups by day 14 post-feeding.

Our last prediction was that mice that received a higher dose of virus would mount a more robust neutralizing antibody response. This prediction was supported, with significantly lower PRNT_80_ values in mice fed upon by one mosquito than mice fed upon by more than one mosquito. Furthermore, at the level of the individual mouse there was a remarkably tight and significant association between dose of virus delivered via forced salivation and PRNT_80_ two weeks post-feeding. PRNT_80_ values were not significantly associated with the total number of mosquitoes that fed upon each mouse (ranging from one to nine mosquitoes per mouse), indicating that the association between estimated virus dose and PRNT_80_ values was not confounded by the volume of saliva delivered to the mouse.

Several caveats to this study must be acknowledged. First, in our test of the transmission-clearance trade-off we did not measure transmission to mosquitoes from infected mice directly. Instead, our test of the transmission–clearance trade-off relied on the assumption that higher ZIKV viremias would result in higher transmission to mosquitoes. This assumption is well supported by previous studies in the literature [20], including our own studies using A129 mice and *Ae. albopictus* mosquitoes [21]. Second, we were not able to measure clearance, since ZIKV was not cleared in the five-day period over which we measured viremia. Instead, we analyzed PRNT_80_ values as an indication that an immune response was mounting to clear the virus. Althouse et al. [40] reviewed studies of dengue virus dynamics following experimental delivery of the virus to non-human primates by needle and found that increasing dose of virus was associated with shorter lag to viremia and shorter duration of viremia. Longer-term studies of ZIKV replication in juvenile A129 mice would be fruitful but are logistically difficult due to the limited amount of blood that can be drawn from these small animals. Third, the transmission–clearance trade-off focuses on the role of the immune system in clearance, but we utilized an immunodeficient mouse model, chosen for its sensitivity to ZIKV infection [29]. This model did allow us to track viremia, disease and adaptive immune responses in an animal system following mosquito feeding. Successful infection of immunocompetent mice with ZIKV typically requires artificially blunting the type-I interferon response or adapting the virus [41]. None of these models can fully recapitulate ZIKV infection in a human. For this reason, nonhuman primates are used as more relevant model for human infection and disease. A recent study showed that the course of ZIKV infection in macaques differed when the virus was delivered by the bite of five infected *Ae. aegypti* mosquitoes compared to needle inoculation [25]. This study found that peak virus titer was delayed following delivery via mosquito bite compared to needle delivery, similar to what we report here in A129 mice. However, it did not attempt to vary the number of mosquitoes delivering virus, nor were enough monkeys infected to analyze the impacts of variation in dose delivered by five mosquitoes.

Even with these caveats, our results add to the small but growing body of work supporting the transmission–clearance trade-off for vector-borne viruses [20,30]. Future work in natural, immunocompetent hosts, which would be humans or non-human primates in the case of ZIKV, should also pursue the relationship between virus dose, intrinsic and innate immune responses, and within-host virus dynamics. Directly extrapolating our observations to human infections, a single mosquito bite may be sufficient to transmit ZIKV to a susceptible human, and the subsequent dynamics of infection likely depend on both the number of mosquito bites that initiate infection and the dose of virus delivered by each mosquito. The low threshold for ZIKV infection also highlights the critical importance of individual protection from mosquitoes through methods such as repellents, appropriate clothing, screens and bed nets [42,43,44,45].

## Figures and Tables

**Table 1:** Mosquito feeding, mouse infection and seroconversion outcomes.

ID # (Gen-der)	Original Treatment Group (no. Mosquitoes/Carton)	No. Mosquitoes that Fed	No. Mosquitoes with Detectable Virus in Saliva (Summed Virus Titer log_10_ffu)	Final Treatment Group (Used for Statistical Analysis)	Mouse Viremia (log_10_ffu/mL Serum) on Designated Day Post-Feeding. Greyed out Cells Indicate that Mouse Was Not Sampled on that Day.					PRNT_80_ at Day 14 Post-Feeding
					1	2	3	4	5	
1 (M)	1	1	1 (1.1)	1	3.32		4.52		5.60	40
2 (M)	1	1	1 (1.23)	1	<LOD ^1^		4.95		5.32	20
3 (M)	1	1	1 (0.70)	1	<LOD		<LOD		<LOD	<20
4 (F)	1	0	NA ^2^	Excluded		<LOD		<LOD		<20
5 (F)	1	1	0	1		3.59		6.38		20
6 (F)	1	1	1 (0.48)	1		3.38		5.38		20
7 (M)	2	2	1 (2.79)	2 to 4	3.36		7.15		4.51	ND ^3^
8 (M)	2	2	1 (0.70)	2 to 4	3.65		7.28		4.30	20
9 (M)	2	1	0	Excluded	<LOD		<LOD		<LOD	<20
10 (M)	2	1	1 (1.04)	1		6.49		6.30		40
11 (M)	2	1	0	Excluded		<LOD		<LOD		<20
12 (M)	2	1	1 (2.26)	1		5.38		5.26		40
13 (M)	5	3	1 (2.26)	2 to 4	5.28		6.32		3.62	40
14 (M)	5	4	3 (2.49)	2 to 4	2.85		7.15		4.48	80
15 (M)	5	3	3 (1.80)	2 to 4	4.26		6.04		5.30	80
16 (F)	5	4	2 (2.54)	2 to 4		4.3		5.4		80
17 (F)	5	4	1 (0.70)	2 to 4		6.20		5.43		<20
18 (F)	5	3	3 (1.11)	2 to 4		6.15		5.15		40
19 (M)	10	7	4 (1.83)	6 to 9	2.0		6.59		4.63	20
20 (M)	10	7	4 (1.49)	6 to 9	4.45		6.42		4.63	40
21 (M)	10	6	6 (3.03)	6 to 9	3.49		7.08		4.15	160
22 (F)	10	8	6 (2.74)	6 to 9		6.49		5.38		80
23 (F)	10	9	3 (1.36)	6 to 9		5.30		5.62		40
24 (F)	10	8	4 (2.38)	6 to 9		6.11		5.58		80

^1^ LOD: limit of detection = 0.9 ffu/mL.^2^ N/A: not applicable.^3^ ND: not done.
